# One Hundred and Sixty Six Cases of Cancer of the Pregnant Uterus Occurring Since 1886

**Published:** 1896-08

**Authors:** George H. Noble

**Affiliations:** Atlanta, Ga.


### ONE HUNDRED AND SIXTY-SIX CASES OF CANCER OF THE PREGNANT UTERUS OCCURRING SINCE 1886.*

BY GEORGE H. NOBLE, M.D.,
ATLANTA, GA.

*(Concluded from last number.)*

There were three forceps deliveries with previous operations, two by incisions and one curetting. Severe bleeding occurred in the two cases of incisions, one mother dying in twelve hours, and in the other case the child was lost. The remaining case was successful, also an additional one without a previous operation. Estimated results in such a small collection are very uncertain, but, as far as they go, they bear out the claim that the use of the forceps is attended with some danger, chiefly from rupture of the diseased cervix and as a carrier of infection. They also support the claim of Baudelocque† that 75 per cent. of mothers and 50 per cent. of children recover. (See Table XI.)

I have encountered but one instance of amputation of the anterior lip during labor, resulting in the recovery of the mother and death of the child.

At five different times tumors were removed intra-partum by

* Read before the Southern Surgical and Gynecological Association in November, 1895.
† Taken from Charpentier's "Cyclopedia of Obstetrics and Gynecology," vol. iii., p. 168.

scissors, curette, forceps, thermocautery, etc., with four immediate recoveries of both mother and child. The case that died was a tumor of the cervix and vagina, which was partially removed; mortality 20 per cent. (See Table XII.)

Incision of the cervix is a subject that is likely to present itself for consideration in a great many cases at or near the end of gestation. It is therefore one of considerable importance to the child and also to the mother, as it may be the means of evacuating the uterus preparatory to a subsequent extirpation. Seven cases with five immediate recoveries (mortality 28.5 per cent.) are shown in this list. Hemorrhage is the great danger to be feared; the two deaths in the list are chargeable to it, dying respectively in two and twelve hours. Another case was forcibly dilated by the hand, and the uterus ruptured through into the bladder and peritoneal cavity. She lived for three and one-half months, and is therefore on the list of immediate recoveries. Two others died respectively in five weeks and in two years. The final results were death in all the cases fully recorded. Several had protracted confinements, as follows: one was in labor thirty-six hours; one in labor one and one-half days; one in labor six days; one in labor eight days. To this is ascribed in a very large measure the death of three babies. The infantile mortality was 35 per cent. After incisions the babies were delivered as follows: four by turning, with one death; one by extraction, with one death; one by forceps, with one death; two spontaneously, with one death. Of the seven cases, four were dead within three and one-half months after labor. It appears that after the cutting the hemorrhage is so great that there is a demand for immediate delivery or plugging of the womb with the extremities of the child for the purpose of controlling it. (See Table XIII.)

Five induced abortions have been encountered, with a mortality of 20 per cent., the death in this case being due to puerperal fever. One case had a carcinoma the size of a hazelnut, successfully removed on the seventh day of childbed. The woman conceived again, and was delivered three years later at full term. One uterus had a deep rupture of the cervix, to which the curette and cautery were applied, resulting in normal childbed. The ultimate results are unknown, except in the one case which was cured by removal of the tumor and was well at the end of three years. As abortion destroys the child and does not materially benefit the mother, it becomes a question of doubtful utility, especially in cases that are amenable to other methods of treatment. (See Table XIV.)

Lewis,* of New Orleans, states that about 40 per cent. of all cases abort spontaneously.

Gusserow on Cohnstein says it is about 35 per cent., only 32 per cent. of the children being born alive, and hardly 20 per cent. lived until their mothers left the bed. "Here we have 20 per cent. of living children, and one-half of them without mothers."

The expectant plan of treatment presents a very good showing —that is, twenty-one cases with nineteen recoveries, two deaths, making a mortality of 10.5 per cent., including five cases under seven months of gestation, or 14.2 per cent. by excluding the five latter cases. Of the twenty-one cases the disease was confined to the cervix or a portion of the same, only two of which were extensive, leaving fifteen cases limited in extent; three of the remaining had invaded the neighboring tissues, and one was not stated. Thus the comparatively low mortality is explained, for a like number of cases would have shown a much more woful set of figures.

Charpentier† states that in forty-seven cases twelve died of rupture of the uterus and three of laceration of the cervix, or 31.9 per cent. Chantreuil places the maternal mortality at 36.7 per cent., and the writer at 24.3 per cent, the average of which is 30.4 per cent, childbed mortality.

After confinement 35 per cent died within three months of cancer, 28.5 per cent. of others had recurrences, while the remainder were not observed; thus 64.4 per cent. were either dead or in a helpless condition soon after childbed.

Of the sixteen recoveries among the cases advanced to seven months of gestation, nine succumbed to the disease, one died of an operation, and six are not reported; thus no final cures are to be found in the list of those treated by the expectant plan. (See Table XV.)

*Charpentier's "Cyclopedia of Obstetrics and Gynecology," vol. iii., p. 166.
†Charpentier's "Encyclopedia of Obstetrics and Gynecology," vol. iii., p. 167.

The very good showing has changed into a very poor one.

Chantreuil places the infantile mortality at 60 per cent., Cohnstein at 57 per cent., Hermann at 40 per cent., the writer at 50 per cent., making an aggreate mortality of 51.8 per cent.

Cohnstein (Bar thesis) states that 68 per cent. go to full term, and Hermann puts it at 28.3 per cent., which gives an average of 48.1 per cent. Then if only 48.1 per cent. of pregnancies go on to full term and 51.8 per cent. of these die, the estimate of successful issue is 24.8 per cent. of all the pregnancies in the cancerous uterus.

The best way to arrive at a conclusion as to the most satisfactory method of conducting a case of this sort is first to exclude all the operative measures that have resulted in high death-rate and accomplish but little good. Artificial abortion secures to the mother very little reduction in the childbed mortality, and defers death only for a limited time. When it is done with a view to subsequent extirpation the advantages gained in the reduction of the size of the womb are counterbalanced by loss of valuable time and puerperal fever; and as the uterus can be extirpated as safely during pregnancy as at other times, artificial abortion is worse than useless. So also should amputations and partial amputations of the cervix be discarded if any hope for the mother remains. It is true that a few cases have been cured by this means, but the number is so small that it will not pay for the chance it has thrown away for saving the mother's life. In like manner all "dilly-dally" methods, such as curetting and cutting off the exuberant growths, should be eschewed as dangerous despoilers of time and opportunity. The next consideration is whether we should act in the interest of the mother or the child, or both. In the incipiency of the disease, when the mother's chances are good, give her the benefit of it. Late in the disease, when the mother's case is hopeless, look to the interest of the child. Between these will be found cases of doubt in which there will be found room for the exercise of judgment. Careful perusal of the statistics will show that vaginal hysterectomy is the most satisfactory means of securing permanent relief in the early period of gestation. Next to it is abdominal hysterectomy in suitable cases. The former gives an immediate mortality of 4 per cent.

and an ultimate recovery of 33.3 per cent. at the end of two years, but the conceptions are all destroyed. So we have here a comparison, upon the one hand, of 33.3 per cent. of ultimate recoveries of the mothers under vaginal hysterectomy, and, upon the other hand, 20 per cent. of ultimate recoveries of the children under the expectant treatment, which proves the former decidedly preferable.

At the close of gestation, when the mother's case is hopeless, she should be delivered by such means as will best serve the child's interest, though her immediate safety and comfort should not be disregarded. In instances of partial obstruction of the cervix incision may answer; when the obstruction is complete, Cæsarean section is indicated. Of the three methods, the conservative gives the best results, the infantile mortality being 11.5 per cent., against 50 per cent. of the expectant treatment. Or, out of sixty mixed operations forty-four children were born alive (70.3 per cent.), showing that any of the operative measures for delivering the fetus is superior to the expectant treatment.

In the doubtful cases the fetus may be near the period of viability and the mother's chance hopeful. In that case gestation might be continued until the child is viable, when the uterus should be evacuated and afterwards removed. This opinion is sustained by the success attained in operations done in the puerperal state, all the hysterectomies recovering.

Thus, a short summary shows that vaginal hysterectomy should be safe in the early months of pregnancy and the puerperal state, when there is a reasonable hope for the mother.

The abdominal hysterectomy should be done under the above conditions when the uterus is too large to be rapidly and safely removed through the vagina.

That at or near the end of pregnancy Cæsarean section (conservative) should be resorted to when the child's interest is to be considered.

That Cæsarean section with Freund's operation is permissible when the disease is confined to the uterus and the child viable.

That in doubtful cases cutting of the cervix and rapid delivery may be judicious when the incision can be made in unulcerated or non-infiltrated tissue.

That as there are four chances to one against the life of the fetus, and as an equal or greater number of mothers may be ultimately cured in the early stages of the disease, the safety of the fetus should not be allowed to hazard the life of the mother.

And that, upon the other hand, the futile efforts directed to the interest of the mother when her case is hopeless should not jeopardize the safety of the fetus in the latter months of pregnancy.

186 *South Pryor Street.*

